# Clinical and Radiological Characterization of the Long-Term Association between Unaffected/Minimally Affected Implants and History of Severe Periodontitis: A Retrospective Study

**DOI:** 10.3390/diagnostics13111880

**Published:** 2023-05-27

**Authors:** Darian Rusu, Viorelia Rădulescu, Stefan-Ioan Stratul, Ionut Luchian, Horia Calniceanu, Octavia Vela, Simina Boia, Giorgios Kardaras, Salvatore Chinnici, Andrada Soanca

**Affiliations:** 1 Department of Periodontology, Faculty of Dental Medicine, Anton Sculean Research Center for Periodontal and Peri-Implant Diseases, “Victor Babes” University of Medicine and Pharmacy, 300041 Timisoara, Romania; rusu.darian@gmail.com (D.R.); horia_calniceanu@yahoo.com (H.C.); octavia_vela@yahoo.com (O.V.); drsalvatorechinnici@gmail.com (S.C.); 2 Department of Periodontology, Faculty of Dental Medicine, “Grigore T. Popa” University of Medicine and Pharmacy, 16 Universitatii Street, 700115 Iasi, Romania; 3 Department of Periodontology, Faculty of Dental Medicine, Applicative Periodontal Regeneration Research Unit, Iuliu Hatieganu University of Medicine and Pharmacy, 400012 Cluj Napoca, Romania

**Keywords:** implants, minimally affected implants, progressive periodontitis, unaffected implants

## Abstract

Objectives: The objectives of this research were to compare, retrospectively, the clinical and radiographic modifications of periodontal parameters and peri-implant conditions and to analyze the relationship between the changes in periodontal parameters and peri-implant conditions over a mean follow-up period of 7.6 years in a treated population with progressive/uncontrolled periodontitis and at least one unaffected/minimally affected implant. Materials and methods: Nineteen partially edentulous patients having 77 implants inserted, with a mean age of 54.84 ± 7.60 years, were matched for age, gender, compliance, smoking status, general health, and implant characteristics. Periodontal parameters were evaluated in the remaining teeth. Means per teeth and implants were used when making comparisons. Results: Statistically significant differences were observed between baseline and final examination in teeth for tPPD, tCAL and MBL. Furthermore, at 7.6 years, statistically significant differences existed between implants and teeth with regard to iCAL and tCAL (*p* = 0.03). Multiple regression analyses were performed and revealed a significant association regarding iPPD and CBL with smoking and periodontal diagnosis. In addition, FMBS was significantly associated with CBL. Unaffected/minimally affected implants were found more frequently in the posterior mandible, with longer lengths (>10 mm) and small diameters (<4 mm), including in screwed multi-unit bridges. Conclusions: The study results appear to reflect minimally affected mean crestal bone-level loss around implants in comparison to the marginal bone-level loss around teeth when exposed to uncontrolled severe periodontal disease over a mean period of observation of 7.6 years, while the unaffected/minimally affected implants seemed to benefit from a combination of clinical factors, including posterior mandibular position, smaller diameters, and screwed multi-unit restorations.

## 1. Introduction

Periodontitis and peri-implantitis are caused by a dysbiosis produced in the multiple species biofilm [1,2,3,4,5]. An inter-dependent relationship occurs between biofilm accumulation and the inflammation of peri-implant tissues [6]. It has been shown that sterile oral implants are colonized by microbes within half an hour after exposure to the oral cavity environment [7]. Furthermore, studies from the literature show that periodontal pathogens from periodontally compromised teeth colonize the newly inserted implants [8,9,10,11,12].

The authors of a review of the literature about the factors influencing dental implant failures from 2014 identified primary failures (implants that never osseointegrate) and secondary failures (which are the majority of cases and are preceded by crestal bone loss [13]. Microbiological and/or biomechanical challenges [10,14,15] and peri-implantitis [16,17] seem to be linked to implant failure.

Potential risk factors for peri-implantitis, with considerable evidence in the literature, have been identified. There is a large body of data indicating that patients with periodontitis are at a higher risk for developing peri-implantitis [18,19,20,21,22,23,24,25]. Also, poor oral hygiene and absence of compliance have been identified as risk indicators for peri-implantitis [23,24,26,27,28,29,30]. Over time, smoking has been significantly associated with chronic periodontitis, with attachment and tooth loss [31,32], and with peri-implantitis [18,20,33,34]. The available evidence is still inconclusive [27] regarding the association between diabetes and peri-implantitis [25,28,33,35].

**Figure 1:**
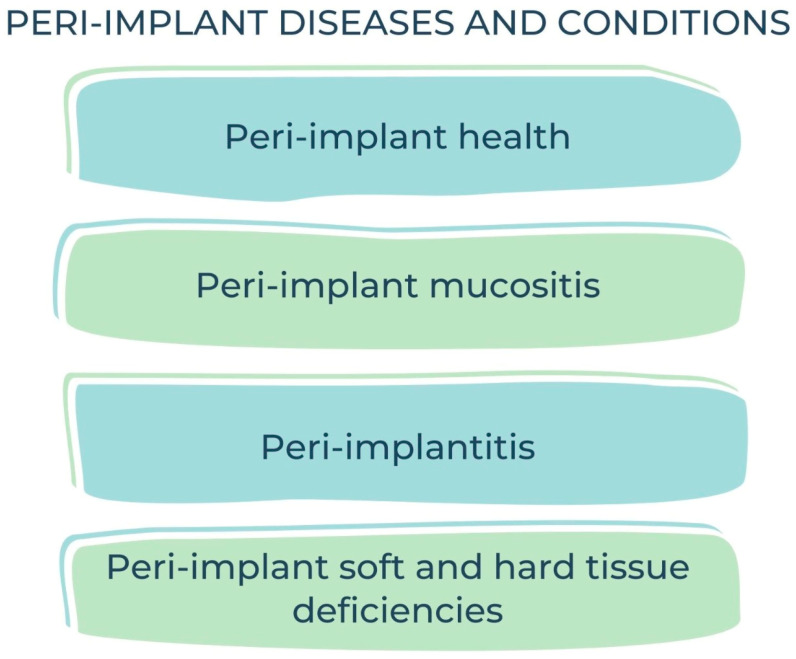
Current classification of peri-implant diseases and conditions.

The latest World Workshop (2017) on the Classification of Periodontal and Peri-Implant Diseases and Conditions clearly separated peri-implant mucositis and peri-implantitis as the two categories of peri-implant diseases [36,37] (Figure 1).

The queries were whether or not fully or partially edentate periodontally compromised patients rehabilitated using dental implants to support fixed dental prostheses [38,39,40] present an increased risk of developing peri-implantitis than patients without a history of periodontitis, what the dimension of the risks are, and how they can be enhanced by therapy with implants [41]. The authors of a systematic review reported that patients with a history of periodontitis showed increased probing depths, greater peri-implant bone loss, and a higher incidence of peri-implantitis. In addition, an acceptable implant survival rate was reported in these individuals attending supportive periodontal treatment (SPT) [38]. It appears that a history of treated periodontitis does not influence short term implant survival rates [42]. However, a reduced number of patients may be refractory to periodontal treatment, continue to lose periodontal attachment and teeth during SPT, and also appear to have a higher incidence of implant complications and failures [43]. Conversely, the results of a study from 2001 show that progressive periodontitis does not always entail an increased risk for peri-implantitis [44].

Although some studies showed a significantly increased radiographic marginal bone loss around implants in periodontally compromised patients compared with periodontally healthy patients [45,46,47,48], there are studies that failed to show any correlation between radiographic bone loss around dental implants and the alteration of periodontal status around teeth in patients experiencing periodontal disease progression [44,49]. Moreover, recent clinical and radiological observations in patients with severe (stage III and IV) periodontitis surprisingly showed that a substantial number of implants seem to be unaffected/minimally affected over a long period of time, despite continuous degradation of the periodontal support [45,50].

At this point, a pertinent question regarding implant treatment in patients treated/un-treated for periodontitis is whether patients showing an alteration in periodontal status may also have an increased risk for peri-implant disease development. The aims of this retrospective study were to analyze the clinical and radiographic modifications of periodontal parameters and peri-implant conditions in a treated population with progressive/uncontrolled periodontitis and at least one unaffected/minimally affected implant over a mean observation period of 7.6 years of SPT. Furthermore, the association between changes in periodontal parameters and peri-implant conditions was investigated.

## 2. Materials and Methods

This retrospective research was approved by the Ethical Committee of Scientific Research of the Victor Babes University of Medicine and Pharmacy, Timisoara (approval no. Nr. 67/16.12.2022), respecting ethical principles of research on humans and data confidentiality.

As we proceeded for a previous retrospective study [50], this retrospective study was built from a database of patients treated for generalized Stage III-IV periodontitis between 1999–2023. The desired dimension of the study population was reached (19 subjects) by consecutively selecting the subjects meeting the inclusion criteria. The conservative standard active periodontal therapy (APT) and SPT were performed in a private practice in Timisoara, Romania.

The clinical and radiographic records were used for data extraction and the clinical diagnosis was retrospectively formulated according to the New Classification for Periodontal and Peri-implant Diseases and Conditions (2018) by the same periodontist (VR) [51,52]. Patients were enrolled if they met the following inclusion criteria: (1) diagnosed stage III and IV periodontitis, grade B, C [51,52,53] (2) had/had not regular SPT performed (3) progressive periodontitis after initial implant therapy, (4) ≥18 years old, (5) presence of at least one implant inserted prior APT (before presentation for periodontal treatment) or after APT, and (6) presence of at least one unaffected/minimally affected implant. Exclusion criteria were: (1) patients with immune systemic disease (e.g., HIV) (2) bisphosphonate administration, and (3) uncontrolled diabetes.

Smoking habits were recorded in terms of current exposure (cigarettes/day) and patients were grouped as follows: light smokers (<10 cigarettes/day), moderate smokers (<20 cigarettes/day) and heavy smokers (≥20 cigarettes/day) [54,55].

### 2.1. Case Definitions

According to Tonetti et al. (2018), a subject was considered a periodontitis case if: “(1) interdental clinical attachment loss (CALoss) was detectable at ≥2 non-adjacent teeth, or (2) buccal or oral CALoss ≥ 3 mm with pocketing > 3 mm is detectable at ≥2 teeth” [51]. Uncontrolled progression of periodontitis was defined according to Tonetti et al. (2005) as the presence of ≥2 teeth showing a longitudinal proximal CALoss of ≥3 mm between two subsequent periodontal evaluations. If longitudinal interdental clinical attachment level measurements were not available, interdental radiographic bone loss was assessed.

The case definitions of peri-implant health, peri-implant mucositis and peri-implantitis were established according to Berglundh et al. (2018). A case of peri-implant health was defined in the absence of: “clinical signs of inflammation, bleeding (BOP) and/or suppuration (SUP) on gentle probing, increase in implant pocket depth (iPPD) compared to previous examination, crestal bone loss beyond crestal bone level (CBL) changes resulting from initial bone remodeling”. The diagnosis of peri-implant mucositis required: BOP ± SUP on gentle probing, ±increase of iPPD compared to previous examinations and absence of CBL beyond crestal bone-level changes resulting from initial bone remodeling [56]. A case of peri-implantitis was defined by the following criteria: BOP and/or (SUP), increase in iPPD compared to previous examinations and presence of CBL. If previous examination data were not available, the diagnosis of peri-implantitis was required: with BOP and/or SUP on gentle probing, iPPD of ≥6 mm, and/or CBL ≥ 3 mm apical of the most coronal portion of the intraosseous part of the implant [56].

### 2.2. Clinical Evaluation

During periodontal evaluations in SPT, clinical parameters were measured and recorded in patients’ periodontal chart. Periodontal pocket depths (PPD) and recession (REC) were measured to the nearest millimeter at six sites per tooth using PCP-UNC15 probes (Hu-Friedy, Chicago, IL, USA). In this study, the term tPPD was used when referring to PPD measured in teeth and iPPD was used for PPD measured in implants. Clinical attachment level (CAL) was assessed as being the distance from the cemento-enamel junction/restoration margin to the most coronal limit of the epithelial attachment. Additionally, tCAL was used for CAL measured in teeth, and iCAL was used when referring to implants. Bleeding on probing (BOP) and plaque were assessed dichotomously at six sites per tooth. SPT recall intervals were established according to the periodontal risk-assessment tool [48].

For implants, the following outcomes were assessed and recorded: (1) BOP, (2) peri-implant SUP and/or fistula, (3) iPPD at the six sites using Williams Colorvue^®^ probes (Hu-Friedy, Chicago, IL, USA).

### 2.3. Patient’s Chart Evaluation

Patient’s charts served as data sources for the following patient-related characteristics: age, gender, compliance, smoking status, and systemic diseases (diabetes, autoimmune). Patients were classified into regular compliers (RCs) and irregular compliers (ICs), according to Costa et al. [57,58]: RCs were considered to be patients attending 100% of the recommended recall interval while ICs were those who missed any scheduled visits but continued attending SPT.

### 2.4. Radiographic Examination

**Figure 2:**
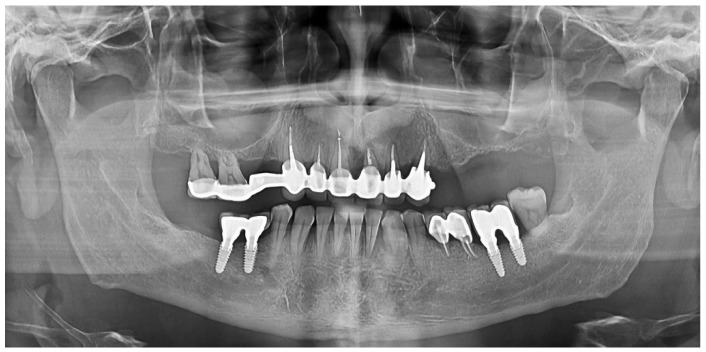
Patient’s radiograph at the beginning of SPT.

**Figure 3:**
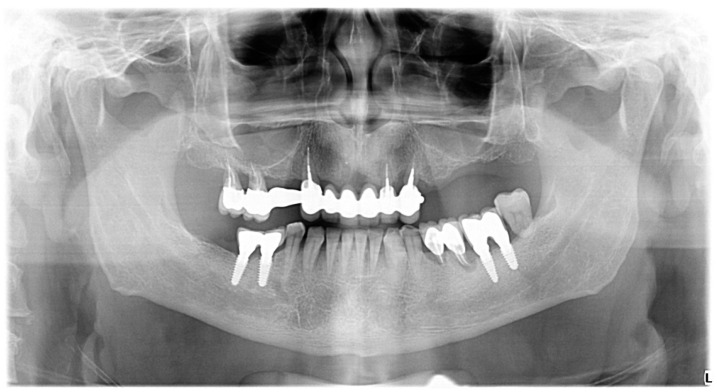
Unaffected implants (reg. 3.6, 3.7, 4.5, 4.6) in a patient with progressive periodontitis.

Radiographic examination was performed on orthopantomograms (e.g., Figure 1 and Figure 2). The dental software Instrumentarium Cliniview^TM^ (Palodex Group Oy Nahkelantie 150, Tuusula, Finland) was used for taking and importing digital radiographs which were analyzed on a large (24′) computer screen. The distance from the implant shoulder to the alveolar bone crest was measured in millimeters at the mesial and distal aspects using the software’s measuring tools [59]. A bone-loss mean value per patient was calculated using the mesial and distal measurements resulting from the crestal bone level (CLB). The mean value per patient of the marginal bone level (MBL) was calculated by measuring, on radiographs, the distance between the CEJ/restoration margin—radiographic bone level at mesial and distal site of each tooth. Unaffected implants were considered implants with the bone level reaching the implant shoulder both on mesial and on distal sites, as viewed on radiographs. Minimally affected implants were considered implants in which the bone level reached one millimeter below the implant shoulder, as viewed on the radiographs (Figure 2 and Figure 3).

### 2.5. Calibration

Intra- and inter-calibration sessions were performed by the three examiners (RV, SIS, RD) on 10 individuals who were not part of the population for this study and who had at least one restoration supported by dental implants. The mean intra-examiner calibration was 0.87 and the mean inter-examiner calibration was 0.85, suggesting an acceptable accordance with the intra-class correlation coefficient (applied before to standardize data acquisition and study variable assessment) [59]. All radiographic assessments were performed by the same examiner, already calibrated for a previous study (BM) [50].

### 2.6. Statistical Analysis

For clinical parameters CAL and PPD, the full mouth plaque score (FMPS), and full mouth bleeding score (FMBS), a full mouth mean was calculated. Additionally, for tooth-level (tCAL, tPPD, MBL) and implant-level (tPPD, tCAL, CBL) clinical and radiographic parameters, a mean value per patient and per teeth or implants was calculated. For statistical analyses, only smoking status at baseline was used. Means, standard deviations (SD), frequency and percentages were used for expressing data distributions. Normality of the distribution of the parametric data was assessed using a Shapiro–Wilk Test. For continuous data, intra- and inter-group comparisons were done using Student’s t-test. The types of tests used are mentioned in each table’s footnotes. Multiple backward regression analyses were used to identify periodontal and patient factors influencing the peri-implant tissue status at seven years. All *p*-values < 0.05 were considered statistically significant. For the statistical analysis MedCalc^®^ Statistical Software version 20.218 (MedCalc Software Ltd., Ostend, Belgium; https://www.medcalc.org, accessed on 3 February 2023) was used.

## 3. Results

### 3.1. Sample Description

The baseline characteristics of the patients and implants are presented in Table 1, Table 2 and Table 3; statistically significant differences were not observed.

12 (63.15%) subjects were women (F) and 7 (36.84%) subjects were men (M) (Table 1). Subjects’ ages ranged from 46 to 76, and the mean age was 54.84 ± 7.60 years. The majority of the patients (63.15%) attended regularly SPT recall appointments. The follow-up mean interval was 7.68 ± 7.14 years. Regarding smoking status, four (21.05%) patients were heavy smokers, five (26.31%) were moderate smokers and 10 (52.63%) were non-smokers. The distribution of baseline periodontal diagnosis is shown in Table 1. Regarding the systemic status, three patients (15.78%) reported hypertension and ischemic heart disease, one (5.26%) patient reported an autoimmune disease, and one patient reported diabetes mellitus type II (5.26%).

The characteristics of all implants analyzed are depicted in Table 2 in terms of frequency and percentages.

Table 3 presents the characteristics of the minimally affected/un-affected implants in the study population. Seven (36.84%) implants were identified in the maxilla, while 12 (63.15%) implants were identified in the mandible. Regarding the position of insertion, 15 (78.94%) implants were located in the posterior region (premolar, molar) and four (21.05%) implants were inserted in the incisive-canine region. Six (31.57%) implants met on the opposite arch to a natural tooth, four (21.05) implants met a bridge on implants, five implants met a bridge on natural teeth, three (15.78%) implants met removable dental prostheses and only one (5.26%) implant met, on the opposite arch, a removable prosthesis supported by implants (overdenture). The mesial neighbor was represented by a tooth for 13 (68.4%) implants and by an implant for the rest of the six (31.6%) minimally affected/unaffected implants. The corresponding values for the distal neighbor was a tooth in six (33.3%) cases and the remaining 12 (66.7%) cases were implants. In some patients, the preparation of the receiving site needed bone augmentation techniques for a proper insertion of the implant, involving the following procedures: guided bone regeneration (GBR) was performed for eight (42.1%) unaffected/minimally affected implants, while sinus lifting was performed for the insertion of two (10.5%) of the unaffected/minimally affected implants.

### 3.2. Clinical Findings

Table 3 shows the overall periodontal parameters (both teeth and implants) at baseline examination and at final examination. A statistically significant difference (*p* = 0.0016) was obtained only for FMBS. The increase in FMBS was from 25.47 ± 14.22 at baseline to 44.63 ± 19.87, with a variation of 19.16 ± 17.27. Even if mean PPD did not reach a statistically significant value, the results showed that mean CAL was marginally statistically significant (*p* = 0.05).

As can be seen in Table 4, statistically significant differences regarding pocket depth and attachment level were found only for teeth. The mean increase in tPPD was 0.75 ± 0.52 mm (*p* = 0.001), ranging from 3.03 ± 0.37 at baseline to 3.78 ± 0.64 upon final examination. The corresponding statistically significant data for the tCAL was 0.71 ± 1.03 mm (*p* = 0.04), ranging from 3.86 ± 0.95 at baseline to 4.57 ± 1.11 upon final examination. Regarding implants, a mean non-statistically significant iPPD increase of 0.36 ± 0.04 (from 3.17 ± 0.52 at baseline to 3.53 ± 0.75 at final examination) was recorded. The difference between baseline and final examination for iCAL during the same observation period was 0.37 ± 0.66 mm. Moreover, statistically significant differences were obtained during inter-group comparison for iCAL and tCAL both at baseline (*p* = 0.01) and upon final examination (*p* = 0.003). (Table 5)

### 3.3. Radiographic Findings

Table 6 shows that the CBL loss around the implants was 0.3 ± 0.8 mm, ranging from 0 mm to 2.7 mm at baseline and from 0 mm to 3 mm upon final examination. The corresponding statistically significant result for the mean MBL alteration for teeth was 0.74 ± 0.70 (*p* = 0.002), ranging from 2.7 mm to 5.1 mm at baseline and from 3.3 mm to 6.5 mm upon final examination. 

In the present study, smoking (*p* = 0.01) and periodontal diagnosis (*p* = 0.00) were found to be significantly correlated to pocket depths at implant sites (Table 7).

In addition, FMPS, smoking and periodontal diagnosis were statistically significant corelated with mean crestal bone level (CBL) at implant sites (Table 8).

## 4. Discussion

“Marginal bone loss around implants is in the great majority of cases associated with immune-osteolytic reactions. Complicating factors include patient genetic disorders, patient smoking, cement or impression material remnants in the peri-implant sulcus, bacterial contamination of the implant components and technical issues such as loose screws, mobile components or fractured materials” [60]. The hypothesis that periodontitis progression involves an increased susceptibility for periimplantitis is based on several studies [61,62], hence this study investigated implants with a mean follow-up period of 7.68 ± 4.17. The case definition of peri-implant diseases has a great variability between studies. We used for this study the latest case definition adopted by the 2017 World Workshop on the Classification of Periodontal and Peri-Implant Diseases and Conditions [56].

Our retrospective study investigated clinical changes and radiographic marginal and crestal bone changes in a population consisting in 19 patients who, despite comprehensive periodontal treatment in a periodontal practice, followed by SPT, continued to experience periodontal attachment loss as a result of progressive periodontitis.

In this study’s population, the prevalence of peri-implantitis was 23.27%, which is comparable to the results of a study from 2005 where the authors reported a prevalence of peri-implantitis of 27.8% [63] and to the results of an another study from 2011, in which a prevalence of peri-implantitis of 30.1% in patients with at least one residual pocket ≥ 6 mm [64] was reported.

The overall mean increase in PPD (teeth and implants) during a mean follow-up period of 7.68 ± 4.17 years was 0.66 ± 0.72 mm. A statistically marginally significant attachment loss of 0.68 ± 1.04 was obtained for overall mean CAL during the same period of observation (*p* = 0.05). In addition, the increase in FMBS during the observation period reached a statistically significant value (*p* = 0.0016).

Statistically significant values were obtained regarding the degradation of the following investigated tooth-related parameters: tPPD, tCAL and mean MBL. The increase (difference to baseline) in tPPD was 0.75 ± 0.52 mm (*p* = 0.001), ranging from 3.03 ± 0.37 at baseline to 3.78 ± 0.64 upon final examination. This value is larger than the one reported in another study investigating the associations between periodontal and peri-implant conditions (0.10 mm) [65]. In addition, the difference between the tCAL value recorded at baseline and the tCAL value recorded upon final examination was greater compared with the aforementioned study (0.71 mm vs. 0.30 mm). Nevertheless, it should be kept in mind that our population showed progressive periodontitis, so an increased alteration of the periodontal parameters at the tooth level compared with the population assessed in the aforementioned study is justified.

The difference between mean MBL in teeth at baseline and upon final examination (ranging from 2.7 mm to 5.1 mm at baseline and from 3.3 mm to 6.5 mm upon final examination) was 0.7 mm, thus reaching a statistically significant value (*p* = 0.002). The resulting marginal bone loss in our population is similar with the one provided by another study on periodontitis-susceptible patients during a follow-up period of 10 years (a mean of 0.59 mm in mesial sites and 0.69 in distal sites) [65].

Conversely to tPPD alteration, a mean non-statistically significant iPPD increase of 0.36 ± 0.04 (from 3.17 ± 0.52 at baseline to 3.53 ± 0.75 upon final examination) was recorded for implants. These findings are comparable with the results of another study which reported an increase in mean iPPD from 2.81 ± 0.77 mm at one year after insertion to 3.13 ± 0.97 mm at three years [66]. At the same time, the results are in opposition to those reported in another study where mean iPPD followed a decreasing tendency to over three years [67]. The differences in follow-up periods may have generated these discrepancies.

In the present study, the mean crestal bone loss during the observation period (7.68 ± 4.17 years) was 0.3 mm, in line with the data reported in another study from 2004 performed on a cohort of 89 periodontitis-susceptible patients where mean mesially and distally peri-implant bone loss was 0.68 and 0.72 mm, respectively [65]. The ten-year reported results in another study on a population with periodontally compromised patients showed a mean bone loss around implants smaller than 1 mm [23]. These results may suggest that, even in a periodontally compromised population, the mean peri-implant bone loss is very reduced.

The present study has revealed significant associations between smoking and periodontal diagnosis at one side, and pocket depths at implant sites on the other side. This observation regarding smoking is in agreement with the findings of Karoussis et al. (2004) [65] and Lindquist et al. (1997) [68]. In addition, the mean crestal bone level (CBL) at implant sites was significantly associated in our study with FMPS, smoking and periodontal diagnosis.

From this latest observation, which was also made by Quirynen et al. (2001) [44], it is still advisable to keep a reduced level of plaque and to strengthen smoking cessation actions towards patient (repeat step 1 of periodontal treatment according to The EFP S3 level clinical practice guidelines) [69,70]. However, the present study failed to find any association between progressive periodontitis at the tooth level and crestal bone loss at the implant level. In addition, these observations are in agreement with Quirynen et al. (2001) [44] and Nevins and Langer (1995) [71].

In the present study, unaffected/minimally affected implants were found more frequently in the posterior mandible, with longer lengths (>10 mm), with small diameters (<4 mm), including in screwed multi-unit bridges. A hypothesis to explain the resistance of implants to the changes in the oral cavity (during the progression of periodontitis), when compared to the alteration of the periodontal status in teeth, might be that teeth “face” periodontitis for a longer time than implants (which are inserted later). Moreover, the mandibular bone has a higher density than the maxilla. Furthermore, the restoration type (i.e., screwed multi-unit bridges vs. cemented bridges) seems to play a role in the “invulnerability” of these implants.

The present study has limitations. Since this is a retrospective study design on a limited number of patients, the results must be prudently interpreted. Seemingly “invulnerable” implants benefit from an unknown combination of clinical factors. Furthermore, the results of the peri- implant health status were reported at the patient level, which may induce a bias compared with an implant-level analysis. It is also worth mentioning that unaffected/minimally affected implants coexist with unaffected/minimally affected teeth in patients with continuous progression of periodontitis.

Future investigations using a greater sample size and a prospective longitudinal study approach are recommended to identify the true associations between changes in periodontal parameters and peri-implant conditions

## 5. Conclusions

The present data indicated that the crestal bone level around implants showed more stability compared to the marginal bone level around natural teeth when exposed to severe uncontrolled periodontal disease over a mean period of observation of 7.6 years. Smoking and periodontal disease diagnosis were significantly associated with increased periodontal pocket depths at implant sites, while FMPS, smoking and periodontal disease diagnosis were significantly associated with crestal bone loss around implants. The unaffected/minimally affected implants seemed to benefit from a combination of clinical factors, including posterior mandibular position, smaller diameters, and screwed multi-unit restorations.

Thus, our research opens new perspectives in the contemporary clinical approaches for periodontal patients who will be treated using clinical protocols that include dental implants.

## Figures and Tables

**Table 1:** Patients’ characteristics.

	Frequency (*N*)	Percent (%)
Sex (*n*, %)		
F	12	63.15
M	7	36.84
Age (years, mean ± SD)	54.84 ± 7.60	
Follow-up	7.68 ± 4.17	
Smoking Status (*N*, %)		
Heavy smoker	4	21.05
Moderate Smoker	5	26.31
Non-smoker	10	52.63
Compliance		
ICs	7	36.85
RCs	12	63.15
Systemic Disorders (*N*, %)		
Unreported	14	73.68
Cardiovascular	3	15.78
Autoimmune	1	5.26
DM type 2	1	5.26

RCs (regular compliers); ICs (irregular compliers); DM type 2- diabetes mellitus type 2.

**Table 2:** Implants’ characteristics.

	Frequency (*N*)	Percent (%)
Health status		
Health	33	42.85
Mucositis	26	33.76
Peri-implantitis	18	23.37
Region		
Anterior	10	12.98
Posterior	67	87.01
Jaw		
Maxilla	19	24.67
Mandible	58	75.32
Diameter		
<4 mm	51	66.23
≥4 mm	26	33.76
Length		
≤10 mm	29	33.76
>10 m	48	62.33
Prosthese		
Single-crown	6	7.79
Bridge	71	92.20
Retention		
Screwed	40	51.94
Cemented	37	48.05
Implant system		
Megagen	41	53.24
OT Medical	26	33.76
MIS	8	10.38
Sybron	2	2.59

**Table 3:** Characteristics of un-affected/minimally affected implants.

	Frequency (*N*)	Percent (%)
Health status		
Health	7	36.84
Mucositis	8	42.10
Peri-implantitis	2	10.52
Region		
Anterior	4	21.05
Posterior	15	78.94
Jaw		
Maxilla	7	36.84
Mandible	12	63.15
Diameter		
<4 mm	12	63.15
≥4 mm	7	36.84
Length		
≤10 mm	7	36.84
>10 m	12	63.15
Prostheses		
Single crown	3	15.8
Bridge abutment	16	84.2
Prosthesis retention		
Screwed	11	57.89
Cemented	8	42.10
Implant system		
Megagen	9	47.36
OT Medical	6	31.57
MIS	4	21.05
Sybron	1	5.26
Antagonist		
Natural tooth	6	31.57
Bridge on implants	4	21.05
Bridge on natural teeth	5	26.31
Removable prostheses	4	21.05
Mesial neighbor		
Tooth	13	68.4
Implant	6	31.6
Distal neighbor		
Tooth	6	33.3
Implant	12	66.7
Type of site augmentation		
GBR	8	42.1
Sinus lift	2	10.5

**Table 4:** Baseline and final comparison regarding periodontal clinical parameters for the entire population.

	T0	T1	Difference to Baseline	*p*-Value
FMBS	25.47 ± 14.22	44.63 ± 19.87	19.16 ± 17.27	0.0016 *
FMPS	10.10 ± 6.87	14.78 ± 16.00	4.68 ± 3.99	0.24
mean PPD	3.24 ± 0.81	3.90 ± 0.62	0.66 ± 0.72	0.078
mean CAL	3.90 ± 1.21	4.58 ± 0.85	0.68 ± 1.04	0.05

Student *t* test; * Statistically significant; FMBS—full mouth bleeding score, FMPS—full mouth plaque score; overall PPD—PPD mean value measured at teeth and implants; overall CAL—clinical attachment level mean value measured at both teeth and implants.

**Table 5:** Intra-group (a) and inter-group (b) comparison between baseline and final examination for teeth and implants.

	T0	T1	Difference to Baseline	*p*-Value ^a^
mean iPPD	3.17 ± 0.52	3.53 ± 0.75	0.36 ± 0.04	0.09
mean tPPD	3.03 ± 0.37	3.78 ± 0.64	0.75 ± 0.52	0.001 *
*p*-value ^b^	0.3	0.2	0.002 *	
mean iCAL	3.22 ± 0.55	3.59 ± 0.76	0.37 ± 0.66	0.09
mean tCAL	3.86 ± 0.95	4.57 ± 1.11	0.71 ± 1.03	0.04 *
*p*-value ^b^	0.01 *	0.003 *	0.2	-

Student *t* test; * Statistically significant; mean iPPD/iCAL—pocket depths/clinical attachment level mean value measured at implant sites; mean tPPD/tCAL—pocket depths/clinical attachment level mean value measured at teeth sites; ^a^—*p*-value for intra-group comparison; ^b^—*p*-value for inter-group comparison.

**Table 6:** Intra-group comparison between baseline and final evaluation for CBL and MBL.

	T0	T1	Difference to Baseline	*p*-Value
mean CBL	0.6 ± 0.8	0.9 ± 0.9	0.3 ± 0.8	0.28
mean MBL	3.52 ± 0.6	4.26 ± 0.79	0.74 ± 0.70	0.002 *

Student *t* test; * Statistically significant; mean CBL—mean value per group of the distance measured radiographically between implant shoulder and crestal bone at mesial and distal site/implant; mean MBL—mean value per group of the distance measured radiographically between CEJ/restoration margin and marginal bone at mesial and distal site/tooth.

**Table 7:** Multiple linear regression identifying factors that influence iPPD.

Independent Variables	Coefficient	Std. Error	*t*	*p* Value	r_partial_	r_semipartial_	VIF
(Constant)	1.8971						
FMBS	−0.01	0.00	−1.93	0.0739	−0.45	0.29	1.10
PERIO DIAGNOSIS	0.28	0.06	4.56	0.0004 *	0.77	0.69	1.51
SMOKING	−0.96	0.36	−2.66	0.0186 *	−0.57	0.40	2.05
MBL	0.39	0.19	1.97	0.0682	0.46	0.29	1.90

Backward multiple regression analysis; * Statistically significant; FMBS—full mouth bleeding score; MBL—mean teeth bone level.

**Table 8:** Multiple linear regression identifying factors that influence the CBL.

Independent Variables	Coefficient	Std. Error	*t*	*p* Value	r_partial_	r_semipartial_	VIF
(Constant)	1.06						
FMPS	−0.03	0.01	−2.99	0.009 *	−0.62	0.47	1.29
SYSTEMIC	−0.28	0.14	−1.96	0.070	−0.46	0.31	1.16
SMOKING	−1.67	0.42	−3.90	0.001 *	−0.72	0.62	1.54
PERIO DIAGNOSIS	0.36	0.09	4.00	0.001 *	0.73	0.63	1.69

Backward multiple regression analysis; * Statistically significant; FMBS—full mouth bleeding score.

## Data Availability

Data are available from the corresponding author upon reasonable request.
